# 1119. Case Study. Four Points, Zero Skin Adhesive Contact: Securing the Pediatric Burn Airway

**DOI:** 10.1093/jbcr/irag033.400

**Published:** 2026-04-06

**Authors:** Vickie R Walker, Alexis L McQuitty

**Affiliations:** Shriners Children's - Texas, League City, TX; Shriners Children's - Texas, Galveston, TX

## Abstract

**Patient Presentation (age range, injury details, relevant history):**

An 8-year-old previously healthy female sustained 52% TBSA second- and third-degree burns involving the bilateral lower extremities, left upper extremity, and face following a residential house fire. She was orally intubated at an outside hospital for airway protection and transferred to our pediatric burn center. Bronchoscopy confirmed mild inhalation injury. The patient remained intubated in the pediatric intensive care unit (PICU) while awaiting operative excision and debridement.

**Clinical Challenges:**

Traditional ETT fixation methods—adhesive tape, commercial devices, or cloth ties—were contraindicated due to:

• Extensive facial burns and fragile tissue.

• Risk of added skin trauma.

• High likelihood of prolonged intubation due to inhalation injury and TBSA burn severity.

Maintaining a secure, skin-sparing airway was critical to prevent unplanned extubation, minimize reintubation risk, and allow ongoing wound care.

**Management Approach:**

A non-adhesive 4-point fixation technique was employed at bedside upon PICU admission:

• Materials: Standard ICU tape folded onto itself, creating non-adhesive strips.

• Application: Two strips anchored from the crown of the head to the ETT; two additional strips looped beneath the ears and around the head, securing the tube in place.

• Key Advantages: Even tension distribution, no skin adhesive contact, rapid reapplication, and minimal need for specialized equipment.

This technique was maintained throughout her ICU course, including perioperative transfers and multiple dressing changes.

**Outcomes:**

The patient’s airway remained secure and complication-free throughout the duration of oral intubation and mechanical ventilation. There were no episodes of unplanned extubation (UE), no mucosal or skin injury, and the fixation method reliably withstood frequent wound care, Q2 turning, and routine oral care. This approach ensured a consistently safe and stable airway environment, even during high-frequency repositioning and critical nursing interventions, demonstrating the technique’s reliability under intensive care conditions.

**Lessons Learned:**

• The 4-point non-adhesive fixation technique reliably maintained a secure airway throughout prolonged intubation, Q2 turning, oral care, and frequent wound care without unplanned extubation or injury.

• Eliminating adhesive contact protected fragile skin, reducing risk of complications.

• The method’s simplicity, reproducibility, and durability allowed safe airway management during multiple dressing changes.

• This technique is easily taught to multidisciplinary teams, improving consistency and safety in high-acuity pediatric burn care.

**Applicability to Practice:**

This case reinforces the value of the 4-point non-adhesive fixation method as a low-cost, reproducible solution to a complex airway management challenge in pediatric burn care. With a demonstrated UE rate < 1% in our institutional experience spanning three decades, this technique is highly scalable and can be implemented across burn and critical care units to:

• Enhance patient safety.

• Reduce airway-related complications.

• Protect fragile tissue integrity.

• Standardize best practices for pediatric airway security.

